# The impact of COVID-19 on palliative care social work: An online survey by a European Association of Palliative Care Task Force

**DOI:** 10.1177/02692163231167938

**Published:** 2023-04-10

**Authors:** Audrey Roulston, Jana Ross, Patricia Dobrikova, Tania Piccione, Carla Reigada, Marie Mackova, Maria Wasner

**Affiliations:** 1 School of Social Sciences, Education and Social Work, Queen’s University Belfast, Northern Ireland, UK; 2 Queen’s University Belfast and Ulster University, Northern Ireland, UK; 3 Department of Social Work, University of Trnava, Trnava, Slovakia; 4 Regional Home Care Program, SAMOT, Palermo, Italy; 5 Global Observatory of Palliative Care, University of Navarra, Pamplona, Spain; 6 Department of Social Sciences, PRIGO University, Havířov, Czech Republic; 7 Katholische Stiftungshochschule München, München, Germany

**Keywords:** Palliative care, social work, pandemics, COVID-19

## Abstract

**Background:**

The SARS-Cov-2 (COVID-19) pandemic affected the delivery of health and social care services globally. However, little is known about how palliative care social work services were impacted.

**Aim:**

The aim of this study was to capture and analyse data from palliative care social workers who provided professional support in a range of settings across 21 countries during the COVID-19 pandemic.

**Design:**

A cross-sectional survey-based design was used for this empirical study and this paper primarily focuses on the quantitative responses.

**Setting/participants:**

Participants, palliative care social workers, were drawn internationally via members of the EAPC Social Work Task Force and the World Hospice Palliative Care Social Work network.

**Results:**

We received 362 survey responses from 21 countries. Most (79%) respondents worked with adults in in-patient units or hospitals. The number of referrals during COVID-19 increased more in non-European countries, compared to European countries. The full range of social work services could no longer be delivered, existing services changed and 65.3% of participants reported higher levels of pressure during the pandemic, which was linked to higher levels of staff absence and additional duties. For many respondents (40.8%), this included facilitating online communication between patients and their families.

**Conclusions:**

Our findings indicate that restrictions to limit the spread of COVID-19 resulted in adaptations to service delivery, increased pressure on staff and moral distress, like other health and social care professions. All members of the palliative team need support and supervision to ensure effective interdisciplinary working and team cohesion.

**What is already known about this topic?**

- Health and social care services were disrupted by the COVID-19 pandemic, which brought challenges for palliative care services.
- Government restrictions on visiting minimised opportunities for communication between patients, relatives and professionals in palliative care settings.
- Patients were less likely to die with a relative at their bedside due to visitor restrictions.

**What this paper adds?**

- Implementing visitor restrictions caused moral distress, but virtual platforms were utilised to promote communication between patients, relatives and colleagues.
- During the pandemic, existing services changed and new services were created in response to increased pressure on staff.
- Palliative care social workers are experienced and well-educated professionals, whose contribution to interdisciplinary team working may be undervalued.

**Implications for practice, theory or policy**

- Supervision, humanisation of patients and psychological support should be a priority to manage increased workloads, reduce pressure and promote patient-centred care.
- Transitioning to online service delivery and virtual visits highlighted inequity of access to smartphones or the internet but improved efficiency, waiting times, frequency of contact and geographical reach for aspects of service delivery.
- The consequences of restrictions imposed during the COVID-19 pandemic may increase the prevalence of prolonged grief disorder, which will need to be addressed by General Practitioners, mental health practitioners, bereavement support services and through educating individuals, families and communities.

## Background

According to the World Health Organisation,^1^ palliative care aims to improve the quality of life of patients living with a life-threatening illness, and their families. Palliative care social workers are primarily responsible for assessing and intervening in the psychosocial and functional status, emotional and mental health, knowledge of preferences, family coping and bereavement risk, relationships, culture, spirituality and resource needs.^2^

The SARS-CoV-2 coronavirus (COVID-19) pandemic generated life-defining challenges on a global level and posed the greatest threat to people living with pre-existing health conditions, in poverty, the elderly and migrants.^3^ All health and social care workers faced exceptional demands through the crisis,^4^ exacerbated by the risk of infection, governmental restrictions and suffering.^5–7^ Face-to-face contact with medical or nursing staff was hampered by personal protective equipment (PPE). Pivoting to virtual meetings with patients and relatives caused moral distress,^8,9^ or professionals perceived that holistic care was compromised.^10^ According to Hanna et al.^11^ health and social care professionals were confronted with increased patient numbers and reduced staffing levels during the pandemic. Weaver et al.^12^ reported that regardless of where people worked, pivoting to virtual communication weakened team cohesion.

During formal lockdowns, people in hospitals and care homes were isolated from relatives and friends,^13^ with some hospices not allowing visitors for 10–12 weeks.^14^ People living at home faced a restriction of formal and informal support, resulting in some managing complex symptoms or experiencing caregiver burnout.^5^ Relatives found telephone or virtual contact in place of face-to-face visits distressing,^15^ they were seldom able to sit at the bedside or say goodbye in person^16^ and some described the death of their loved one as traumatic.^17,18^ People were confronted with restrictions when planning cremations, burials and funerals^19^ due to limits on how many people who could attend funerals, which may increase the likelihood of prolonged grief disorders.^20^

Negative psychosocial consequences have been described in the context of previous pandemics (i.e. Spanish Flu of 1918, 1957 Asian Flu, 1968 Hong Kong Flu and 2009 H1N1 Swine Flu), with an emphasis on the need for psychosocial support.^21^ The field of hospice and palliative care social work is one key provider of psychosocial care across Europe and more globally.^22^ Due to the associated restrictions, palliative care social workers adapted their practice to maintain contact with patients and relatives through innovative communication modalities and new strategies for intervention.^18,23^ An international collaboration^24^ highlighted how palliative care social workers in Argentina, India, Northern Ireland and Zimbabwe pivoted away from inpatient support to provide outreach, education, consultation, public health information or bereavement follow-up calls following deaths. Based on a survey conducted in the US, there were four central psychosocial challenges that patients and social workers had to face in hospice and palliative care during COVID-19 pandemic: isolation, barriers to communication, disruption of systems and issues related to grieving.^16^

A larger body of literature now exists about the impact of COVID-19 on the mental health of health and social care professionals.^25^ However, given the paucity of research involving palliative care social workers, the current study aims to capture demographic and employment-based data on those who provided professional support across several countries during the COVID-19 pandemic.

## Methods

### Research question

The study aimed to answer the following two research questions:

- What was the impact of COVID-19 on palliative social care workers internationally?
- What are the demographic and employment characteristics of palliative care social workers who worked during the COVID-19 pandemic?

### Design

A cross-sectional survey-based design was used in the current study. The research design is based on the epistemological assumption that palliative care social workers have been affected by the pandemic. An online survey offered an opportunity to gain insight into their experiences. The Checklist for Reporting Results of Internet E-Surveys (CHERRIES) was used to report. The CHERRIES has been designed to improve the reporting of internet-based surveys.^26^ Formal ethical approval for the study was granted in September 2021 by the School of Social Sciences, Education and Social Work Research Ethics Committee in Queen’s University Belfast (SREC107_2021). The study applied ethical principles including written informed consent procedures, voluntary participation, secure storage of data and anonymised data in reports, publications and presentations. Only respondents who provided informed consent, after reading the participant information sheet, were allowed to access the study questionnaire. No personal or identifying information was collected in the study and no incentives were offered for participation. The data custodian, who collated data and conducted the analyses, was independent of the EAPC Social Work Task Force and the World Hospice Palliative Care Social Work network.

### Population

The population for the study involved all palliative care social workers who were registered to receive emails from the EAPC Social Work Task Force and the WHPCSW network. The EAPC Social Work Task Force, established in 2009, consists of 13 palliative care social work practitioners, academics and researchers, aiming to upskill social workers as leaders, practitioners, educators and researchers in palliative care across Europe (https://www.eapcnet.eu/eapc-groups/task-forces/social-worker/).

The WHPCSW network is a global community of palliative care social workers, established in 2020, who share insights and resources for practice, research and education. Potential participants were palliative care social workers in the following countries: Australia, Austria, Brazil, Canada, Czech Republic, England, Germany, Hong Kong, Hungary, Italy, Netherlands, New Zealand, Northern Ireland, Republic of Ireland, Scotland, Slovakia, South America, Spain, Sweden, the United States and Wales. Participants had to be at least 18 years old and working in palliative care during the COVID-19 pandemic. This was determined by self-reporting.

### Setting

The study was conducted entirely online. It was not advertised, an email invitation was sent to all potential participants and those who agreed to take part, completed an online survey, hosted on the web-based Qualtrics platform. Prior to completing the survey, they were presented with the participant information sheet and the consent form.

### Sampling

The study used convenience sampling and the recruitment methods (see section below) ensured that the invitation to take part in the study was sent specifically to palliative care social workers.

### Recruitment

Members of the EAPC Social Work Task Force with leadership roles in their respective countries and co-chairs of the WHPCSW network emailed invitations using membership lists. The online survey was open for participation in October and November 2021.

### Data collection

Ten members of the EAPC Social Work Task Force were involved in identifying the research aims, the proposed methods for data collection and the questions to be included in the online survey. The group explored different ways of capturing information by using dichotomous, open ended and multiple-choice questions. Members tested the survey for technical functionality, duration to complete and clarity of questions, given the range of contexts and countries. Redundant questions and options were removed to keep the questionnaire as short as possible. Once consensus was reached, the questionnaire was translated into Czech, English, German, Italian, Slovak, Spanish and Portuguese, to widen participation. The survey contained both quantitative (*n* = 15) and qualitative (*n* = 3) questions spread across nine pages of the survey platform. Number of questions per page differed depending on the length of each question and the number of response options. A back button was provided so respondents could go back and change their response options. All questions were mandatory, but a *Prefer not to say* or *Other – Please specify* options were provided where applicable. The questions captured participants’ demographic data (i.e. age, gender, highest level of education, country); work-related information (i.e. employment status, work setting, years in palliative care); services provided pre-COVID (i.e. population, place and description); and how COVID-19 impacted the number of referrals received, the workforce and service provision. A copy of the questionnaire is provided in Supplemental Materials. It was an open survey, and anyone with the link was able to take part. Due to the anonymous nature of the survey, it was not possible to check the uniqueness of survey visitors. IP addresses were not checked as colleagues could have used the same computer to complete the survey. However, since no incentives were provided for completing the survey, it is unlikely that anyone completed it more than once.

### Data analysis

The quantitative data analysis was conducted in SPSS 26 and includes frequencies and percentages in order to describe the study sample. Valid percentages are provided throughout to account for missing data, as certain respondents left the questionnaire prior to completing it. Since the study is descriptive, missing values were not estimated and the sample size differed for each analysis and is reported in each table. No statistical correction was used to adjust for the non-representativeness of the sample. Due to different sample sizes across countries, direct cross-country comparisons are not reported, but European countries are compared to non-European countries.

Open-ended survey responses, which add important nuances to the quantitative items that precede it, were analysed separately to enhance, confirm or refine the story told through quantitative data. Raw responses were independently reviewed by the first two authors, to begin the process of preliminary data coding using a combination of *deductive codes* drawn from the research questions and *inductive codes* generated by the data. Initial codes were grouped into categories while attributes emerged; forming new codes.^27^ Quotes in the results are provided with participant identification (ID) numbers.

## Results

The survey had a completion rate of 76% (number of people who submitted the last questionnaire page divided by the number of people who started the survey). Three hundred sixty-two survey responses were received from across 21 countries (see Table 1). Most responses came from Italy (*n* = 77), followed by the USA (*n* = 71), Germany (*n* = 33), Czech Republic (*n* = 29) and Austria (*n* = 27). The ‘Other’ category includes countries with fewer than three responses (i.e. Argentina, Hungary, Poland, Portugal, Singapore, Wales and Zimbabwe). As shown in Table 1, most respondents were female (*n* = 309, 85.36%) and educated to a master’s level (*n* = 190, 52.49%). Demographic and work-related information by country is presented in Supplemental Table 1.

**Table 1.** Survey responses by country and demographic and work-related information of the full sample (*N* = 362).

	*n* (%)
Country	
Australia	13 (3.59)
Austria	27 (7.46)
Canada	3 (0.83)
Czech Republic	29 (8.01)
England	15 (4.14)
Germany	33 (9.12)
Italy	77 (21.27)
Netherlands	5 (1.38)
Northern Ireland	14 (3.87)
Republic of Ireland	18 (4.97)
Slovakia	23 (6.35)
Spain	6 (1.66)
Sweden	19 (5.25)
USA	71 (19.61)
Other	9 (2.49)
Demographic and work-related information of the full sample	
Age *M* (SD)	44.57 (11.17)
Gender *n* (%)	
Male	37 (10.22)
Female	309 (85.36)
Other	1 (0.28)
Prefer not to say	15 (4.14)
Highest educational qualification *n* (%)	
Bachelor’s Degree	105 (29.01)
Master’s Degree	190 (52.49)
PhD	19 (5.25)
Other	38 (10.50)
Prefer not to say	10 (2.76)
Employment status *n* (%)	
Employed full-time	226 (62.43)
Employed part-time	94 (25.97)
Self-employed	28 (7.73)
Retired	5 (1.38)
Prefer not to say	9 (2.49)
No. of years working in palliative care *M* (SD)	9.52 (7.90)

Presented are valid percentages.

When asked where they worked during the COVID-19 pandemic, the most frequently selected option in the full sample was *home palliative care service with adults* (*n* = 128; 41.16%), followed by *hospital palliative care support team with adults* (*n* = 86; 27.65%) and *inpatient hospice with adults* (*n* = 72; 23.15%). When comparing European with non-European countries, the most frequently selected options were *home palliative care service for adults* (49.33% of European respondents) and *hospital palliative care support team for adults* (62.79% of non-European respondents) (see Supplemental Table 2).

Respondents were asked about the number of referrals received each month pre-COVID-19 and how this change during the pandemic. In the full sample, 33.96% responded that the number pre-COVID was between 0 and 10 referrals per month. A further 26.10% received between 11 and 20 referrals, 14.47% of the sample received between 21 and 30 referrals and the rest of the sample received more than 30 referrals per month (see Supplemental Table 3). When comparing responses from European and non-European respondents, 79.92% of European respondents and 58.23% of non-European respondents received up to 30 referrals each month, prior to COVID-19. Compared to the time before COVID-19, 41.19% of respondents in the full sample reported that the number of referrals to their social work service during COVID-19 was *about the same*. A further 16.98% reported it was *lower* and for the remaining 41.82% of respondents, it was *little* or *much higher* (see Supplemental Table 4). The number of referrals each month was *about the same* for 43.51% of respondents in European countries compared 34.18% in non-European countries. More respondents in non-European countries reported an increase in the number of referrals to the palliative care social work service than in the European countries (53.16% vs 38.08% respectively).

Respondents were also asked ‘*How did COVID-19 affect you or other staff involved in providing palliative care social work services or bereavement support in your organisation?*’ The full sample responses are shown in Table 2 and the breakdown by country is provided in Supplemental Table 5. The most frequent responses in the full sample were *Increased pressure on staff due to nature of COVID-19 restrictions* (65.27%), *Using IT for online meetings or appointments* (54.34%), *Working from home* (45.34%), *Facilitating online communication between patients and families or friends* (40.84%) and *Increased pressure on staff due to increased number of clients* (36.98%). Over a quarter of the sample were *redeployed* (16.4%), *furloughed* (10.29%) or *made redundant* (1.93%). There were higher levels of *staff absence due to illness* (36.01%) compared to *staff absence due to caring for dependents* (24.76%). In both European (60.09%) and non-European (80.77%) countries, the most frequently selected option was *Increased pressure on staff due to nature of COVID-19 restrictions*.

**Table 2.** Responses to the question ‘How did COVID-19 affect you or other staff involved in providing palliative care social work services or bereavement support in your organisation?’ (*n* = 311).

Response	*n* (%)
Social work staff were furloughed	32 (10.29)
Social work staff worked from home	141 (45.34)
Social work staff were made redundant	6 (1.93)
Social work staff redeployed	51 (16.40)
Fundraising events were cancelled or postponed	69 (22.19)
New fundraising events/appeals were introduced	14 (4.50)
Higher levels of staff absence due to illness	112 (36.01)
Higher levels of staff absence due to caring for dependants	77 (24.76)
Increased pressure on staff due to increased number of clients	115 (36.98)
Increased pressure on staff due to additional duties	142 (45.66)
Increased pressure on staff due to nature of COVID-19 restrictions	203 (65.27)
Purchasing new IT equipment to work remotely	77 (24.76)
Using IT for online meetings/appointments	169 (54.34)
Educating patients/carers for online meetings/appointments	101 (32.48)
Facilitating communication online between patients and families/friends	127 (40.84)
Increased need for supervision or staff support	87 (27.97)
Positive team dynamics/cohesion	68 (21.86)
Better work-life balance	28 (9.00)
Increased understanding of the social work role	52 (16.72)
Increased respect of the social work role	68 (21.86)
Other	14 (4.50)

Reported are valid percentages to account for missing data. Multiple response options were allowed.

When asked how COVID-19 affected the social work support provided, almost half of the respondents said *We could no longer deliver the full range of social work services* (*n* = 135, 46.08%) and *We changed existing social work services* (*n* = 134, 45.73%). About a third of respondents *introduced new social work services* (n = 102, 34.89%), almost a quarter made *Other changes that affected their services or support* (*n* = 70, 23.89%) and a further 24.23% (*n* = 71) selected none of the above options. Taken together, this reveals the extent of changes to service delivery, which affected the range of services being delivered and the need to introduce new services.

In European countries, the most frequently selected response option was *We could no longer deliver the full range of social work services* (47.25%) and in non-European countries it was *We could no longer deliver the full range of social work services* and *We changed existing social work services* (both 42.67%) (see Supplemental Table 6 for a full breakdown of responses by country).

Qualitative responses to the open-ended question ‘How has COVID-19 affected the social work support you provide?’ offer rich insights into changes to the delivery of palliative care social work services. These included social work support, assessments, and counselling pivoting to online, or telephone and family meetings no longer being facilitated (ID44), family centre closures (ID222) or the suspension of bereavement support groups and memorial services (ID168). Some suggested that stopping face-to-face support to people with COVID-19 and their loved ones (ID254), and shifting contact with relatives to the telephone ‘increased stress’ (ID301). Furthermore, ‘very sick patients or older people had a hard time with technical equipment’ (ID72) and were disadvantaged when video or telephone calls replaced face-to-face contact between patients, relatives or social workers.

On a positive note, one respondent was ‘able to provide all the same services as prior to COVID-19, but virtually, including counselling sessions, bereavement support groups, memorials and workshops’ (ID59). Six respondents felt the pandemic led to improvements as they ‘learned new technology and skills to provide Telehealth psychotherapy for patients in an outpatient clinic setting’ (ID188), or that services had been ‘strengthened through tools incorporating telematics networks’ (ID141).

Concerning the virtual delivery of bereavement support and appointments, some found it ‘impersonal’ (ID102) with ‘difficulties due to missing non-verbal aspects’ of communication (ID11). Others reported that offering telephone interviews effectively reduced the waiting time between referral and assessment (ID203), enabled social workers to ‘reach people virtually at a distance that we couldn’t serve before’ (ID48), made better use of resources and improved their work-life balance (ID244). Some suggested that working remotely increased the intensity of the work by generating more appointments following the removal of travel time (ID52), whereas another respondent believed that this freed them up for more direct service time (ID48).

When asked, ‘*who they provided palliative care social work services to before the COVID-19 pandemic*’ the full sample responses are shown in Table 3, with the breakdown by country in Supplemental Table 7. *Adults with a life-limiting illness* was the most frequently selected option (82.46%), followed by *family carers of patients with a life-limiting illness* (71.93%) and *bereaved relatives or friends* (53.51%). This pattern was largely similar across most countries. In European countries, the most frequently selected response option was *adults with a life-limiting illness* (81.96%) and in non-European countries it was *adults with a life-limiting illness* and *family carers of patients with a life-limiting illness* (both 83.91%).

**Table 3.** Responses to the question ‘Who did you provide palliative care social work services to before the COVID-19 pandemic?’ (*n* = 342).

Response	*n* (%)
Children with a life-limiting illness	66 (19.30)
Young people with a life-limiting illness	102 (29.82)
Adults with a life-limiting illness	282 (82.46)
Family carers of patients with a life-limiting illness	246 (71.93)
Bereaved relatives or friends	183 (53.51)
Staff support/supervision for social workers	88 (25.73)
Staff support/supervision for other members of the MDT	91 (26.61)
Staff training internally	98 (28.65)
Education externally	89 (26.02)
Fundraising	15 (4.39)
Public relations	38 (11.11)
Other	28 (8.19)

Reported are valid percentages to account for missing data. Multiple response options were allowed.

Table 3 also demonstrates the diversity of the social work role within palliative care (i.e. working with patients, families or staff, fundraising and providing training). Positive comments highlight increased respect and recognition for palliative care social workers (ID335), particularly when interventions were ‘indispensable’ (ID30) or when positive feedback from relatives who received counselling reached medical colleagues, who previously viewed this as a ‘last resort’ (ID105). Others felt that palliative care social work had been ‘eliminated’ (ID31), ‘degraded’ (ID311) or ‘forgotten’ (ID151).

COVID-19 restrictions changed service delivery, which increased pressure on staff. Some services were suspended, but most pivoted to virtual or telephone contact. For some, virtual working eliminated travel time and speeded up assessments. It also enabled social workers to reach people in remote areas and for some, it improved work-life balance.

### Discussion

#### Study population

Our full sample consisted of 362 palliative care social workers, the majority of whom were women of working age (44.5 years). This reflects the social work profession, which is female-dominated in terms of direct care and academia^28^ and it is in line with observations from other countries, where during COVID-19, professional women played a central role in frontline care, providing physical, emotional and economic responses to those in need.^4^

#### Changes to service provision

Prior to COVID-19, the participants in our study provided a diverse range of services, reflecting the roles and responsibilities traditionally undertaken within the social work profession.^28,29^ Half of our respondents reported that they were no longer able to provide the full range of social work provision, and existing services changed in response to government restrictions for infection control. For some of our respondents, the cessation of face-to-face contact, and the transition to virtual or telephone communication with service users, families and colleagues was professionally challenging and increased stress, which mirrors other studies.^7–9,27,30,31^ In contrast, other respondents found that transitioning online prompted new ways of working, which was more effective in terms of geographical reach, time management and assessment times, which was also reported in other studies.^32–34^

#### Increased pressure on staff

Almost half of the European countries represented in our study reported that the number of referrals received during the pandemic remained the same, whereas non-European countries observed an increase. Given the increase in death rate during the pandemic, referrals to bereavement services and waiting lists automatically increased.^20^

Respondents in our sample reported feeling increased pressures due to COVID-19 restrictions, which is in line with other international studies.^30^ Increased work pressures during COVID-19 have been linked with increased mental health problems in the health and social care workforce, which if left unaddressed may lead to burn out.^35,36^ Many respondents reported working from home and using virtual technologies for meetings and appointments, which mirrors findings from Jonas et al.^34^ Although not without limitations, Telehealth became an important skillset during the pandemic, as it enabled social workers to continue offering support and connectedness. Consultations, counselling visits, bereavement support groups and memorial services continued to an extent thanks to Telehealth.^34^ However, as mentioned above, the transition to providing virtual support was challenging for some.

Although social workers in many countries, were considered essential to helping and supporting patients and service users, more than a quarter of our sample were redeployed, furloughed or made redundant during the pandemic. This finding highlights the differences in palliative care social worker role across the globe, or perhaps the different importance given to the profession, which may warrant further examination.

### What this study adds

This study enabled a diverse group of palliative care social workers to articulate their experiences of how the COVID-19 pandemic affected their work. It emphasises the importance of flexibility, communication and interdisciplinary team working, the inequities when services rely on internet access and smartphones, and the consequences for relatives who experienced interruptions to death and funeral rituals.

### Future research needed

Future research should investigate ways to support the workforce through professional recognition, manageable workloads, reduced pressure and regular supervision. It will also be important to examine the benefits and disadvantages of using virtual platforms to deliver services.

### Strengths and limitations of the study

Convenience sampling through existing networks and email lists resulted in a good response rate, but under representation from non-European countries with a larger palliative care workforce (i.e. USA or Canada) and countries where EAPC Task Force members had no links. It is unclear if email invitations went into ‘junk mail’. Convenience sampling also means that the results cannot be considered representative of all palliative care social workers. Another limitation is that we used an open survey and inclusion criteria were that respondents self-reported their occupation as a palliative care social worker. However, considering that the email invitations were sent through the EAPC Social Work Task Force and the WHPCSW network mailing lists, it is unlikely that respondents were from other occupational groups. It is possible that some respondents participated more than once, but this is unlikely considering that no incentives were offered for participation.

### Conclusion

This study used survey data from a total of 362 palliative care social workers from across 21 countries. It provides rich insights into the impact of the COVID-19 pandemic on palliative care social workers and the services provided. Over 40% of respondents in our sample reported an increase in referrals during the pandemic, compared to the time before. Almost two-thirds reported that they felt increased pressures due to the COVID-19 restrictions and over a half started using virtual technology for meetings and appointments. The study also found that palliative care social workers were innovative. They adapted existing service provision and developed new services to offer continuous support to their patients. It is unclear if revised service provision or virtual meetings will remain longer-term.

## Supplemental Material

sj-pdf-1-pmj-10.1177_02692163231167938 – Supplemental material for The impact of COVID-19 on palliative care social work: An online survey by a European Association of Palliative Care Task Force Click here for additional data file. Supplemental material, sj-pdf-1-pmj-10.1177_02692163231167938 for The impact of COVID-19 on palliative care social work: An online survey by a European Association of Palliative Care Task Force by Audrey Roulston, Jana Ross, Patricia Dobrikova, Tania Piccione, Carla Reigada, Marie Mackova and Maria Wasner in Palliative Medicine
