# Supplementary material for: The impact of COVID-19 on palliative care social work: An online survey by a European Association of Palliative Care Task Force
Source: Palliat Med. 2023 Apr 10;37(6):884–92. doi: 10.1177/02692163231167938 (PMC10186133; doi:10.1177/02692163231167938)
Supplement: sj-pdf-1-pmj-10.1177_02692163231167938 – Supplemental material for The impact of COVID-19 on palliative care social work: An online survey by a European Association of Palliative Care Task Force [file sj-pdf-1-pmj-10.1177_02692163231167938.pdf]

Supplementary material

The Impact of Covid-19 on Palliative Care Social Work: An online survey by a European Association of Palliative Care (EAPC) Task Force

Supplementary Table 1

Demographic and work-related information of the full sample (N = 362) and by country

	Country							
	**Full sample (*N* = 362)**	**Australia (*n* = 13)**	**Austria (*n* = 27)**	**Canada (*n* = 3)**	**Czech Republic (*n* = 29)**	**England (*n* = 15)**	**Germany (*n* = 33)**	**Italy (*n* = 77)**
**Age**								
Min / Max / Median	20 / 75 / 45	27 / 68 / 51	32 / 67 / 48	50 / 57 / 54	26 / 61 / 42	32 / 75 / 55	20 / 61 / 42.50	26 / 66 / 38.50
Mean (SD)	44.57 (11.17)	51.23 (11.42)	48.52 (9.64)	53.67 (3.51)	41.52 (10.43)	53.75 (12.44)	42.16 (11.90)	40.41 (10.92)
Prefer not to say *n* (%)	28 (7.73)	0 (0)	4 (14.81)	0 (0)	6 (20.69)	3 (20.00)	1 (3.03)	3 (3.90)
**Gender** *n* (%)								
Male	37 (10.22)	1 (7.69)	5 (18.52)	0 (0)	2 (6.90)	0 (0)	4 (12.12)	10 (12.99)
Female	309 (85.36)	11 (84.62)	20 (74.07)	3 (100.00)	23 (79.31)	15 (100.00)	29 (87.88)	65 (84.42)
Other	1 (0.28)	0 (0)	0 (0)	0 (0)	0 (0)	0 (0)	0 (0)	0 (0)
Prefer not to say	15 (4.14)	1 (7.69)	2 (7.41)	0 (0)	4 (13.79)	0 (0)	0 (0)	2 (2.60)
**Highest educational qualification** *n* (%)								
Bachelor's Degree	105 (29.01)	4 (30.77)	7 (25.93)	2 (66.67)	9 (31.03)	8 (53.33)	15 (45.45)	23 (29.87)
Master's Degree	190 (52.49)	5 (38.46)	8 (29.63)	1 (33.33)	8 (27.59)	6 (40.00)	4 (12.12)	48 (62.34)
Ph.D.	19 (5.25)	3 (23.08)	3 (11.11)	0 (0)	1 (3.45)	0 (0)	1 (3.03)	2 (2.60)
Other	38 (10.50)	1 (7.69)	8 (29.63)	0 (0)	8 (27.59)	1 (6.67)	13 (39.39)	2 (2.60)
Prefer not to say	10 (2.76)	0 (0)	1 (3.70)	0 (0)	3 (10.34)	0 (0)	0 (0)	2 (2.60)
**Employment status** *n* (%)								
Employed full-time	226 (62.43)	5 (38.46)	2 (7.41)	2 (66.67)	23 (79.31)	6 (40.00)	21 (63.64)	44 (57.14)
Employed part-time	94 (25.97)	7 (53.85)	23 (85.19)	1 (33.33)	2 (6.90)	7 (46.67)	36.36)	3 (3.90)
Self-employed	28 (7.73)	1 (7.69)	0 (0)	0 (0)	0 (0)	1 (6.67)	0 (0)	26 (33.77)
Retired	5 (1.38)	0 (0)	1 (3.70)	0 (0)	1 (3.45)	1 (6.67)	0 (0)	2 (2.60)
Prefer not to say	9 (2.49)	0 (0)	1 (3.70)	0 (0)	3 (10.34)	0 (0)	0 (0)	2 (2.60)
**No. of years working in palliative care**								
Min / Max / Median	0* / 39 / 7	1 / 34 / 5	1 / 30 / 12	20 / 21 / 21	1 / 39 / 6	1 / 35 / 13	1 / 27 / 7.25	0* / 35 / 8
Mean (SD)	9.52 (7.90)	8.23 (8.87)	10.39 (6.12)	20.67 (0.58)	9.45 (8.90)	13.67 (9.41)	9.65 (7.89)	9.20 (7.82)
Missing *n* (%)	20 (5.52)	0 (0)	0 (0)	0 (0)	0 (0)	0 (0)	3 (9.09)	8 (10.39)

	Country							
	**Netherlands (*n* = 5)**	**Northern Ireland**	**Republic of Ireland**	**Slovakia (*n* = 23)**	**Spain (*n* = 6)**	**Sweden (*n* = 19)**	**US (*n* = 71)**	**Other (*n* = 9)**

		(*n* = 14)	(*n* = 18)					
**Age**								
Min / Max / Median	31 / 63 / 55	30 / 58 / 40	34 / 60 / 45	27 / 61 / 45	25 / 52 / 43	25 / 66 / 48	26 / 67 / 47	32 / 66 / 39
Mean (SD)	48.00 (14.11)	42.42 (10.17)	46.22 (7.70)	44.75 (9.32)	40.83 (10.30)	45.33 (12.88)	46.69 (10.60)	41.71 (11.27)
Prefer not to say *n* (%)	0 (0)	2 (14.29)	0 (0)	3 (13.04)	0 (0)	1 (5.26)	3 (4.23)	2 (22.22)
**Gender** *n* (%)								
Male	1 (20.00)	2 (14.29)	1 (5.56)	2 (8.70)	2 (33.33)	3 (15.79)	4 (5.63)	0 (0)
Female	4 (80.00)	12 (85.71)	17 (94.44)	19 (82.61)	3 (50.00)	16 (84.21)	66 (92.96)	6 (66.67)
Other	0 (0)	0 (0)	0 (0)	0 (0)	0 (0)	0 (0)	0 (0)	1 (11.11)
Prefer not to say	0 (0)	0 (0)	0 (0)	2 (8.70)	1 (16.67)	0 (0)	1 (1.41)	2 (22.22)
**Highest educational qualification** *n* (%)								
Bachelor's Degree	4 (80.00)	9 (64.29)	5 (27.78)	2 (8.70)	0 (0)	13 (68.42)	1 (1.41)	3 (33.33)
Master's Degree	1 (20.00)	5 (35.71)	10 (55.56)	15 (65.22)	6 (100.00)	4 (21.05)	66 (92.96)	3 (33.33)
Ph.D.	0 (0)	0 (0)	0 (0)	3 (13.04)	0 (0)	1 (5.26)	4 (5.63)	1 (11.11)
Other	0 (0)	0 (0)	3 (16.67)	1 (4.35)	0 (0)	0 (0)	0 (0)	1 (11.11)
Prefer not to say	0 (0)	0 (0)	0 (0)	2 (8.70)	0 (0)	1 (5.26)	0 (0)	1 (11.11)
**Employment status** *n* (%)								
Employed full-time	1 (20.00)	7 (50.00)	16 (88.89)	17 (73.91)	5 (83.33)	14 (73.68)	58 (81.69)	5 (55.56)
Employed part-time	4 (80.00)	7 (50.00)	2 (11.11)	4 (17.39)	1 (16.67)	5 (26.32)	13 (18.31)	3 (33.33)
Self-employed	0 (0)	0 (0)	0 (0)	0 (0)	0 (0)	0 (0)	0 (0)	0 (0)
Retired	0 (0)	0 (0)	0 (0)	0 (0)	0 (0)	0 (0)	0 (0)	0 (0)
Prefer not to say	0 (0)	0 (0)	0 (0)	2 (8.70)	0 (0)	0 (0)	0 (0)	1 (11.11)
**No. of years working in palliative care**								
Min / Max / Median	3 / 28 / 10	2 / 27 / 8	1 / 23 / 5.50	1 / 38 / 5	2 / 13 / 8.50	1 / 25 / 3	1 / 35 / 7	2 / 35 / 7
Mean (SD)	13.40 (10.38)	9.62 (6.67)	8.97 (7.66)	8.60 (8.72)	8.17 (4.45)	5.36 (5.69)	9.58 (7.69)	10 (10.34)
Missing *n* (%)	0 (0)	1 (7.14)	2 (11.11)	2 (8.70)	0 (0)	1 (5.26)	3 (4.23)	0 (0)

Note. * Less than a year. Presented are valid column percentages.

Supplementary Table 2

Responses to the question 'Where did you work during the COVID-19 pandemic?'

	**Place of work**
	n (%)

	Volunteer hospice service	Home palliative care service	Inpatient hospice	Hospital palliative care support team	Palliative care unit	Day hospice, day care centre	Other
**Full sample* (*n* = 311)**							
Adults	38 (12.22)	128 (41.16)	72 (23.15)	86 (27.65)	56 (18.01)	16 (5.14)	37 (11.90)
Children	9 (2.89)	37 (11.90)	10 (3.22)	12 (3.86)	8 (2.57)	0 (0)	14 (4.50)
**Australia (*n* = 12)**							
Adults	1 (8.33)	3 (25.00)	1 (8.33)	5 (41.67)	5 (41.67)	0 (0)	1 (8.33)
Children	0 (0)	2 (16.67)	0 (0)	0 (0)	0 (0)	0 (0)	0 (0)
**Austria (*n* = 27)**							
Adults	5 (18.52)	14 (51.85)	4 (14.81)	5 (18.52)	9 (33.33)	2 (7.41)	0 (0)
Children	0 (0)	3 (11.11)	0 (0)	0 (0)	0 (0)	0 (0)	0 (0)
**Canada (*n* = 3)**							
Adults	2 (66.67)	3 (100.00)	0 (0)	2 (66.67)	1 (33.33)	0 (0)	1 (33.33)
Children	2 (66.67)	2 (66.67)	0 (0)	1 (33.33)	1 (33.33)	0 (0)	1 (33.33)
**England (*n* = 14)**							
Adults	3 (21.43)	5 (35.71)	7 (50.00)	3 (21.43)	1 (7.14)	2 (14.29)	2 (14.29)
Children	2 (14.29)	2 (14.29)	2 (14.29)	0 (0)	1 (7.14)	0 (0)	1 (7.14)
**Germany (*n* = 31)**							
Adults	9 (29.03)	5 (16.13)	8 (25.81)	1 (3.23)	4 (12.90)	0 (0)	4 (12.90)
Children	2 (6.45)	2 (6.45)	1 (3.23)	0 (0)	1 (3.23)	0 (0)	2 (6.45)
**Italy (*n* = 75)**							
Adults	10 (13.33)	49 (65.33)	23 (30.67)	4 (5.33)	14 (18.67)	2 (2.67)	7 (9.33)
Children	1 (1.33)	12 (16.00)	2 (2.67)	0 (0)	2 (2.67)	0 (0)	3 (4.00)
**Netherlands (*n* = 4)**							
Adults	0 (0)	0 (0)	1 (25.00)	2 (50.00)	1 (25.00)	0 (0)	1 (25.00)
Children	0 (0)	0 (0)	0 (0)	0 (0)	0 (0)	0 (0)	0 (0)
**Northern Ireland (*n* = 12)**							
Adults	0 (0)	4 (33.33)	1 (8.33)	2 (16.67)	2 (16.67)	4 (33.33)	3 (25.00)
Children	0 (0)	0 (0)	0 (0)	0 (0)	0 (0)	0 (0)	0 (0)
**Republic of Ireland (*n* = 17)**							
Adults	1 (5.88)	14 (82.35)	8 (47.06)	6 (35.29)	5 (29.41)	3 (17.65)	3 (17.65)
Children	0 (0)	3 (17.65)	1 (5.88)	1 (5.88)	1 (5.88)	0 (0)	1 (5.88)
**Slovakia (*n* = 21)**							
Adults	2 (9.52)	8 (38.10)	8 (38.10)	1 (4.76)	0 (0)	3 (14.29)	1 (4.76)
Children	1 (4.76)	5 (23.81)	0 (0)	0 (0)	0 (0)	0 (0)	2 (9.52)
**Sweden (*n* = 19)**							
Adults	0 (0)	11 (57.89)	4 (21.05)	6 (31.58)	7 (36.84)	0 (0)	2 (10.53)
Children	0 (0)	3 (15.79)	1 (5.26)	0 (0)	2 (10.53)	0 (0)	0 (0)
**US (*n* = 69)**							

Adults	3 (4.35)	9 (13.04)	7 (10.14)	47 (68.12)	7 (10.14)	0 (0)	12 (17.39)
Children	0 (0)	1 (1.45)	3 (4.35)	9 (13.04)	0 (0)	0 (0)	4 (5.80)
**Other (*n* = 7)**							
Adults	2 (28.57)	3 (42.86)	0 (0)	2 (28.57)	0 (0)	0 (0)	0 (0)
Children	1 (14.29)	2 (28.57)	0 (0)	1 (14.29)	0 (0)	0 (0)	0 (0)

	**Place of work** *n* (%)					
	**Hospice / palliative care unit (acute, complex patients)**	**Nursing home / long term residences**	**Hospital palliative care support team**	**Community palliative care service**	**Day hospice, day care centre**	**Other**
**Spain (*n* = 6)**						
Adults	3 (50.00)	1 (16.67)	2 (33.33)	1 (16.67)	0 (0)	0 (0)
Children	1 (16.67)	0 (0)	1 (16.67)	0 (0)	0 (0)	0 (0)
**Other (*n* = 2)**						
Adults	2 (100.00)	0 (0)	2 (100.00)	0 (0)	0 (0)	0 (0)
Children	0 (0)	0 (0)	0 (0)	0 (0)	0 (0)	0 (0)

	**Place of work** *n* (%)						
	**Hospice / palliative care unit**	**Day hospice care**	**Outpatient hospice service**	**Home palliative care services**	**Nursing home / long term residences**	**Hospital services**	**Others**
**Czech Republic (*n* = 29)**							
	8 (27.58)	0 (0)	1 (3.45)	4 (13.79)	13 (44.83)	5 (17.24)	3 (10.34)

Note. Reported are valid country percentages to account for missing data. *n* represents the number of respondents who answered the question. Multiple response options were allowed. Full sample does not include responses from Czech Republic, Spain and one of the 'Other' countries, in which different response options were provided.

Supplementary Table 3

Approximate number of referrals to social work services each month before COVID-19 (n = 318)

	**Number of referrals before COVID-19**											
**Country** *n* (%)	**0 - 10**	**11 - 20**	**21 - 30**	**31 - 40**	**41 - 50**	**51 - 60**	**61 - 70**	**71 - 80**	**81 - 90**	**91 - 100**	**101+**	**Missing response** *n*
Full sample	108 (33.96)	83 (26.10)	46 (14.47)	21 (6.60)	13 (4.09)	7 (2.20)	2 (0.63)	4 (1.26)	3 (0.94)	4 (1.26)	27 (8.49)	44
Australia	3 (23.08)	4 (30.77)	2 (15.38)	2 (15.38)	0 (0)	1 (7.69)	0 (0)	0 (0)	0 (0)	0 (0)	1 (7.69)	-
Austria	7 (28.00)	13 (52.00)	3 (12.00)	1 (4.00)	0 (0)	0 (0)	0 (0)	0 (0)	0 (0)	1 (4.00)	0 (0)	2
Canada	0 (0)	3 (100.00)	0 (0)	0 (0)	0 (0)	0 (0)	0 (0)	0 (0)	0 (0)	0 (0)	0 (0)	-
Czech Republic	20 (68.97)	5 (17.24)	4 (13.79)	0 (0)	0 (0)	0 (0)	0 (0)	0 (0)	0 (0)	0 (0)	0 (0)	-
England	2 (16.67)	3 (25.00)	3 (25.00)	2 (16.67)	1 (8.33)	1 (8.33)	0 (0)	0 (0)	0 (0)	0 (0)	0 (0)	3
Germany	12 (44.44)	7 (25.93)	5 (18.52)	1 (3.70)	2 (7.41)	0 (0)	0 (0)	0 (0)	0 (0)	0 (0)	0 (0)	6
Italy	27 (40.91)	11 (16.67)	7 (10.61)	2 (3.03)	2 (3.03)	2 (3.03)	0 (0)	0 (0)	1 (1.52)	2 (3.03)	12 (18.18)	11
Netherlands	4 (80.00)	0 (0)	0 (0)	0 (0)	0 (0)	0 (0)	1 (20.00)	0 (0)	0 (0)	0 (0)	0 (0)	-
Northern Ireland	6 (50.00)	2 (16.67)	2 (16.67)	2 (16.67)	0 (0)	0 (0)	0 (0)	0 (0)	0 (0)	0 (0)	0 (0)	2
Republic of Ireland	0 (0)	6 (40.00)	4 (26.67)	2 (13.33)	0 (0)	1 (6.67)	0 (0)	0 (0)	0 (0)	0 (0)	2 (13.33)	3
Slovakia	13 (61.90)	6 (28.57)	1 (4.76)	1 (4.76)	0 (0)	0 (0)	0 (0)	0 (0)	0 (0)	0 (0)	0 (0)	2
Spain	2 (33.33)	0 (0)	0 (0)	0 (0)	2 (33.33)	0 (0)	0 (0)	1 (16.67)	0 (0)	0 (0)	1 (16.67)	-
Sweden	4 (25.00)	5 (31.25)	2 (12.50)	1 (6.25)	2 (12.50)	1 (6.25)	0 (0)	1 (6.25)	0 (0)	0 (0)	0 (0)	3
US	4 (6.67)	16 (26.67)	12 (20.00)	7 (11.67)	4 (6.67)	0 (0)	1 (1.67)	2 (3.33)	2 (3.33)	1 (1.67)	11 (18.33)	11
Other	4 (50.00)	2 (25.00)	1 (12.50)	0 (0)	0 (0)	1 (12.50)	0 (0)	0 (0)	0 (0)	0 (0)	0 (0)	1

Note. Reported are valid row percentages to account for missing data.

Supplementary Table 4

Change in the number of referrals to social work services during COVID-19 (n = 318)

	**Number of referrals during COVID-19 compared to the period before COVID-19**				
**Country** *n* (%)	**Lower than before COVID-19**	**About the same**	**A little higher than before COVID-19**	**Much higher than before COVID-19**	**Missing response** *n*
Full sample	54 (16.98)	131 (41.19)	72 (22.64)	61 (19.18)	44
Australia	0 (0)	7 (53.85)	4 (30.77)	2 (15.38)	-
Austria	6 (24.00)	15 (60.00)	3 (12.00)	1 (4.00)	2
Canada	0 (0)	0 (0)	1 (33.33)	2 (66.67)	-
Czech Republic	4 (13.79)	17 (58.62)	5 (17.24)	3 (10.34)	-
England	2 (16.67)	4 (33.33)	2 (16.67)	4 (33.33)	3
Germany	6 (22.22)	18 (66.67)	1 (3.70)	2 (7.41)	6
Italy	10 (15.15)	18 (27.27)	21 (31.82)	17 (25.76)	11
Netherlands	1 (20.00)	2 (40.00)	0 (0)	2 (40.00)	-
Northern Ireland	5 (41.67)	1 (8.33)	2 (16.67)	4 (33.33)	2
Republic of Ireland	3 (20.00)	3 (20.00)	8 (53.33)	1 (6.67)	3
Slovakia	1 (4.76)	11 (52.38)	7 (33.33)	2 (9.52)	2
Spain	0 (0)	3 (50.00)	0 (0)	3 (50.00)	-
Sweden	3 (18.75)	11 (68.75)	2 (12.50)	0 (0)	3
USA	9 (15.00)	19 (31.67)	16 (26.67)	16 (26.67)	11
Other	4 (50.00)	2 (25.00)	0 (0)	2 (25.00)	1

Note. Reported are valid row percentages to account for missing data.

Supplementary Table 5

Responses to the question 'How did COVID-19 affect you or other staff involved in providing palliative care social work services or bereavement support in your organisation?' (N = 311)

		**Country**						
**Response** *n* (%)	**Full sample (*N* = 311)**	**Australia (*n* = 12)**	**Austria (*n* = 25)**	**Canada (*n* = 3)**	**Czech Republic (*n* = 29)**	**England (*n* = 12)**	**Germany (*n* = 27)**	**Italy (*n* = 64)**
Social work staff were furloughed	32 (10.29)	1 (8.33)	4 (16.00)	0 (0)	11 (37.93)	2 (16.67)	1 (3.70)	1 (1.56)
Social work staff worked from home	141 (45.34)	5 (41.67)	16 (64.00)	1 (33.33)	0 (0)	8 (66.67)	14 (51.85)	28 (43.75)
Social work staff were made redundant	6 (1.93)	0 (0)	0 (0)	0 (0)	6 (20.69)	0 (0)	0 (0)	0 (0)
Social work staff redeployed	51 (16.40)	2 (16.67)	3 (12.00)	0 (0)	16 (55.17)	1 (8.33)	2 (7.41)	1 (1.56)
Intensity of the work has changed	209 (67.20)	5 (41.67)	16 (64.00)	3 (100.00)	18 (62.07)	9 (75.00)	22 (81.48)	41 (64.06)
Fundraising events were cancelled or postponed	69 (22.19)	3 (25.00)	4 (16.00)	1 (33.33)	0 (0)	5 (41.67)	11 (40.74)	10 (15.63)
New fundraising events/appeals were introduced	14 (4.50)	0 (0)	0 (0)	0 (0)	0 (0)	2 (16.67)	3 (11.11)	0 (0)
Higher levels of staff absence due to illness	112 (36.01)	5 (41.67)	7 (28.00)	0 (0)	14 (48.28)	4 (33.33)	10 (37.04)	13 (20.31)
Higher levels of staff absence due to caring for dependants	77 (24.76)	4 (33.33)	4 (16.00)	0 (0)	15 (51.72)	5 (41.67)	3 (11.11)	8 (12.50)
Increased pressure on staff due to increased number of clients	115 (36.98)	5 (41.67)	5 (20.00)	3 (100.00)	11 (37.93)	5 (41.67)	2 (7.41)	20 (31.25)
Increased pressure on staff due to additional duties	142 (45.66)	7 (58.33)	6 (24.00)	2 (66.67)	13 (44.83)	7 (58.33)	13 (48.15)	22 (34.38)
Increased pressure on staff due to nature of COVID-19 restrictions	203 (65.27)	10 (83.33)	19 (76.00)	2 (66.67)	14 (48.28)	7 (58.33)	21 (77.78)	28 (43.75)
Purchasing new IT equipment to work remotely	77 (24.76)	3 (25.00)	8 (32.00)	0 (0)	0 (0)	2 (16.67)	10 (37.04)	10 (15.63)
Using IT for online meetings/appointments	169 (54.34)	9 (75.00)	15 (60.00)	2 (66.67)	0 (0)	9 (75.00)	21 (77.78)	25 (39.06)
Educating patients/carers for online meetings/appointments	101 (32.48)	8 (66.67)	1 (4.00)	3 (100.00)	0 (0)	6 (50.00)	7 (25.93)	5 (7.81)

Facilitating communication online between patients and families/friends	127 (40.84)	6 (50.00)	2 (8.00)	2 (66.67)	0 (0)	7 (58.33)	10 (37.04)	14 (21.88)
Increased need for supervision or staff support	87 (27.97)	3 (25.00)	5 (20.00)	2 (66.67)	3 (10.34)	7 (58.33)	6 (22.22)	10 (15.63)
Positive team dynamics/cohesion	68 (21.86)	2 (16.67)	6 (24.00)	0 (0)	1 (3.45)	5 (41.67)	10 (37.04)	9 (14.06)
Better work-life balance	28 (9.00)	0 (0)	3 (12.00)	1 (33.33)	0 (0)	2 (16.67)	2 (7.41)	1 (1.56)
Increased understanding of the social work role	52 (16.72)	2 (16.67)	1 (4.00)	0 (0)	0 (0)	4 (33.33)	4 (14.81)	11 (17.19)
Increased respect of the social work role	68 (21.86)	3 (25.00)	3 (12.00)	1 (33.33)	0 (0)	5 (41.67)	4 (14.81)	16 (25.00)
Other	14 (4.50)	1 (8.33)	3 (12.00)	1 (33.33)	1 (3.45)	1 (8.33)	1 (3.70)	0 (0)

	**Country**							
	**Netherlands (*n* = 3)**	**Northern Ireland (*n* = 12)**	**Republic of Ireland (*n* = 15)**	**Slovakia (*n* = 21)**	**Spain (*n* = 5)**	**Sweden (*n* = 15)**	**US (*n* = 60)**	**Other (*n* = 8)**
Social work staff were furloughed	0 (0)	1 (8.33)	0 (0)	0 (0)	0 (0)	2 (13.33)	7 (11.67)	2
Social work staff worked from home	3 (100.00)	8 (66.67)	7 (46.67)	4 (19.05)	4 (19.05)	7 (46.67)	32 (53.33)	4
Social work staff were made redundant	0 (0)	0 (0)	0 (0)	0 (0)	0 (0)	0 (0)	0 (0)	0
Social work staff redeployed	0 (0)	7 (58.33)	1 (6.67)	0 (0)	4 (19.05)	1 (6.67)	11 (18.33)	2
Intensity of the work has changed	2 (66.67)	7 (58.33)	10 (66.67)	7 (33.33)	2 (9.52)	10 (66.67)	52 (86.67)	5
Fundraising events were cancelled or postponed	0 (0)	2 (16.67)	6 (40.00)	11 (52.38)	0 (0)	3 (20.00)	10 (16.67)	3
New fundraising events/appeals were introduced	0 (0)	0 (0)	3 (20.00)	1 (4.76)	0 (0)	1 (6.67)	1 (1.67)	3
Higher levels of staff absence due to illness	2 (66.67)	5 (41.67)	8 (53.33)	13 (61.90)	1 (4.76)	10 (66.67)	17 (28.33)	3
Higher levels of staff absence due to caring for dependants	1 (33.33)	3 (25.00)	4 (26.67)	9 (42.86)	0 (0)	2 (13.33)	18 (30.00)	1
Increased pressure on staff due to increased number of clients	1 (33.33)	2 (16.67)	6 (40.00)	5 (23.81)	1 (4.76)	6 (40.00)	40 (66.67)	3
Increased pressure on staff due to additional duties	1 (33.33)	4 (33.33)	7 (46.67)	16 (76.19)	3 (14.29)	6 (40.00)	33 (55.00)	2
Increased pressure on staff due to nature of COVID-19 restrictions	2 (66.67)	6 (50.00)	14 (93.33)	16 (76.19)	4 (19.05)	8 (53.33)	48 (80.00)	4

Purchasing new IT equipment to work remotely	2 (66.67)	5 (41.67)	6 (40.00)	5 (23.81)	2 (9.52)	8 (53.33)	13 (21.67)	3
Using IT for online meetings/appointments	3 (100.00)	5 (41.67)	15 (100.00)	5 (23.81)	5 (23.81)	13 (86.67)	40 (66.67)	2
Educating patients/carers for online meetings/appointments	2 (66.67)	3 (25.00)	10 (66.67)	3 (14.29)	1 (4.76)	10 (66.67)	40 (66.67)	2
Facilitating communication online between patients and families/friends	1 (33.33)	6 (50.00)	13 (86.67)	8 (38.10)	2 (9.52)	7 (46.67)	46 (76.67)	3
Increased need for supervision or staff support	2 (66.67)	2 (16.67)	5 (33.33)	10 (47.62)	1 (4.76)	3 (20.00)	26 (43.33)	2
Positive team dynamics/cohesion	2 (66.67)	1 (8.33)	5 (33.33)	5 (23.81)	1 (4.76)	1 (6.67)	19 (31.67)	1
Better work-life balance	1	1 (8.33)	3 (20.00)	1 (4.76)	0 (0)	4 (26.67)	8 (13.33)	1
Increased understanding of the social work role	2 (66.67)	4 (33.33)	3 (20.00)	4 (19.05)	1 (4.76)	2 (13.33)	12 (20.00)	2
Increased respect of the social work role	2 (66.67)	1 (8.33)	6 (40.00)	5 (23.81)	0 (0)	3 (20.00)	17 (28.33)	2
Other	1 (33.33)	1 (8.33)	0 (0)	1 (4.76)	0 (0)	1 (6.67)	2 (3.33)	0

Note. Reported are valid column percentages to account for missing data. Multiple response options were allowed.

Supplementary Table 6 *'How has COVID-19 affected the social work support you provide?' (N = 293)*

		**Country**						
**Response** *n* (%)	**Full sample (*N* = 293)**	**Australia (*n* = 12)**	**Austria (*n* = 23)**	**Canada (*n* = 3)**	**Czech Republic (*n* = 29)**	**England (*n* = 12)**	**Germany (*n* = 22)**	**Italy (*n* = 62)**
We could no longer deliver the full range of social work services	135 (46.08)	8 (66.67)	10 (43.48)	2 (66.67)	9 (31.03)	10 (83.33)	13 (59.09)	19 (30.65)
We changed existing social work services	134 (45.73)	5 (41.67)	11 (47.83)	0 (0)	1 (3.45)	10 (83.33)	16 (72.73)	26 (41.94)
We introduced new social work services	102 (34.81)	4 (33.33)	4 (17.39)	0 (0)	0 (0)	9 (75.00)	10 (45.45)	15 (24.19)
Other changes were made that affected your services or support	70 (23.89)	5 (41.67)	7 (30.43)	2 (66.67)	0 (0)	3 (25.00)	6 (27.27)	14 (22.58)
None of the options	71 (24.23)	1 (8.33)	6 (26.09)	1 (33.33	19 (65.52)	0 (0)	5 (22.73)	19 (30.65)

	**Country**							
**Response** *n* (%)	**Netherlands (*n* = 3)**	**Northern Ireland (*n* = 10)**	**Republic of Ireland (*n* =14)**	**Slovakia (*n* = 18)**	**Spain (*n* = 5)**	**Sweden (*n* = 15)**	**US (*n* = 57)**	**Other (*n* = 8)**
We could no longer deliver the full range of social work services	2 (66.67)	7 (70.00)	11 (78.57)	9 (50.00)	3 (60.00)	9 (60.00)	21 (36.84)	2 (25.00)
We changed existing social work services	1 (33.33)	7 (70.00)	13 (92.86)	5 (27.78)	3 (60.00)	8 (53.33)	26 (45.61)	2 (25.00)
We introduced new social work services	3 (100.00)	3 (30.00)	10 (71.43)	6 (33.33)	2 (40.00)	9 (60.00)	24 (42.11)	3 (37.50)
Other changes were made that affected your services or support	1 (33.33)	3 (30.00)	6 (42.86)	4 (22.22)	2 (40.00)	3 (20.00)	12 (21.05)	2 (25.00)
None of the options	0 (0)	0 (0)	0 (0)	5 (27.78)	0 (0)	0 (0)	12 (21.05)	3 (37.50)

Note. Reported are valid column percentages to account for missing data. Multiple response options were allowed.

Supplementary Table 7

Responses to the question ‘Who did you provide palliative care social work services to before the COVID-19 pandemic?’ (N = 342)

	**Country**							
**Response** *n* (%)	**Full sample (*N* = 342)**	**Australia (*n* = 13)**	**Austria (*n* = 27)**	**Canada (*n* = 3)**	**Czech Republic (*n* = 29)**	**England (*n* = 15)**	**Germany (*n* = 30)**	**Italy (*n* = 69)**
Children with a life-limiting illness	66 (19.30)	2 (15.38)	3 (11.11)	2 (66.67)	5 (17.24)	2 (13.33)	5 (16.67)	16 (23.19)
Young people with a life-limiting illness	102 (29.82)	1 (7.69)	3 (11.11)	2 (66.67)	5 (17.24)	2 (13.33)	6 (20.00)	33 (47.83)
Adults with a life-limiting illness	282 (82.46)	10 (76.92)	23 (85.19)	3 (100.00)	24 (82.76)	14 (93.33)	23 (76.67)	60 (86.96)
Family carers of patients with a life-limiting illness	246 (71.93)	8 (61.54)	24 (88.89)	3 (100.00)	10 (34.48)	14 (93.33)	19 (63.33)	46 (66.67)
Bereaved relatives or friends	183 (53.51)	10 (76.92)	18 (66.67)	3 (100.00)	10 (34.48)	11 (73.33)	17 (56.67)	9 (13.04)
Staff support / supervision for social workers	88 (25.73)	6 (46.15)	5 (18.52)	3 (100.00)	2 (6.90)	7 (46.67)	3 (10.00)	7 (10.14)
Staff support / supervision for other members of the MDT	91 (26.61)	4 (30.77)	10 (37.04)	1 (33.33)	3 (10.34)	6 (40.00)	8 (26.67)	9 (13.04)
Staff training internally	98 (28.65)	3 (23.08)	6 (22.22)	0 (0)	1 (3.45)	9 (60.00)	11 (36.67)	8 (11.59)
Education externally	89 (26.02)	1 (7.69)	11 (40.74)	1 (33.33)	4 (13.79)	4 (26.67)	13 (43.33)	4 (5.80)
Fundraising	15 (4.39)	0 (0)	1 (3.70)	1 (33.33)	2 (6.90)	0 (0)	5 (16.67)	1 (1.45)
Public relations	38 (11.11)	0 (0)	10 (37.04)	0 (0)	2 (6.90)	2 (13.33)	14 (46.67)	3 (4.35)
Other	28 (8.19)	1 (7.69)	2 (7.41)	0 (0)	0 (0)	0 (0)	3 (10.00)	10 (14.49)

	Country							
	**Netherlands (*n* = 5)**	**Northern Ireland (*n* = 13)**	**Republic of Ireland (*n* = 16)**	**Slovakia (*n* = 21)**	**Spain (*n* = 6)**	**Sweden (*n* = 18)**	**US (*n* = 68)**	**Other (*n* = 9)**
Children with a life-limiting illness	0 (0)	0 (0)	9 (56.25)	6 (28.57)	2 (33.33)	2 (11.11)	10 (14.71)	2 (22.22)
Young people with a life-limiting illness	1 (20.00)	0 (0)	5 (31.25)	8 (38.10)	2 (33.33)	8 (44.44)	24 (35.29)	2 (22.22)
Adults with a life-limiting illness	4 (80.00)	13 (100.00)	15 (93.75)	12 (57.14)	3 (50.00)	16 (88.89)	57 (83.82)	5 (55.56)
Family carers of patients with a life-limiting illness	4 (80.00)	12 (92.31)	15 (93.75)	7 (33.33)	5 (83.33)	14 (77.78)	59 (86.76)	6 (66.67)
Bereaved relatives or friends	1 (20.00)	12 (92.31)	15 (93.75)	15 (71.43)	4 (66.67)	13 (72.22)	39 (57.35)	6 (66.67)
Staff support / supervision for social workers	2 (40.00)	6 (46.15)	7 (43.75)	3 (14.29)	1 (16.67)	1 (5.56)	31 (45.59)	4 (44.44)
Staff support / supervision for other members of the MDT	1 (20.00)	3 (23.08)	5 (31.25)	5 (23.81)	1 (16.67)	3 (16.67)	28 (41.18)	4 (44.44)
Staff training internally	1 (20.00)	6 (46.15)	8 (50.00)	3 (14.29)	2 (33.33)	1 (5.56)	34 (50.00)	5 (55.56)
Education externally	1 (20.00)	3 (23.08)	8 (50.00)	3 (14.29)	4 (66.67)	1 (5.56)	27 (39.71)	4 (44.44)
Fundraising	0 (0)	0 (0)	0 (0)	3 (14.29)	0 (0)	1 (5.56)	1 (1.47)	0 (0)
Public relations	0 (0)	0 (0)	0 (0)	4 (19.05)	0 (0)	0 (0)	2 (2.94)	1 (11.11)
Other	0 (0)	2 (15.38)	1 (6.25)	3 (14.29)	1 (16.67)	1 (5.56)	4 (5.88)	0 (0)

Note. Reported are valid column percentages to account for missing data. Multiple response options were allowed.

Supplementary Table 8

Responses to the question 'Before COVID-19, what support did you provide after the death of patients?' (N = 334)

	Country							
**Response** *n* (%)	**Full sample (*N* = 334)**	**Australia (*n* = 13)**	**Austria (*n* = 25)**	**Canada (*n* = 3)**	**Czech Republic (*n* = 29)**	**England (*n* = 15)**	**Germany (*n* = 28)**	**Italy (*n* = 68)**
Immediate post-death support	197 (58.98)	8 (61.54)	17 (68.00)	3 (100.00)	18 (62.07)	13 (86.67)	17 (60.71)	13 (19.12)
Sending information to bereaved individuals / families	198 (59.28)	11 (84.62)	10 (40.00)	3 (100.00)	17 (58.62)	9 (60.00)	10 (35.71)	49 (72.06)
Group support for adults	71 (21.26)	3 (23.08)	6 (24.00)	2 (66.67)	5 (17.24)	5 (33.33)	9 (32.14)	3 (4.41)
Group support for children	25 (7.49)	0 (0)	0 (0)	2 (66.67)	1 (3.45)	3 (20.00)	2 (7.14)	0 (0)
Home visits	107 (32.04)	6 (46.15)	13 (52.00)	2 (66.67)	3 (10.34)	10 (66.67)	12 (42.86)	12 (17.65)
Remembrance services	97 (29.04)	5 (38.46)	9 (36.00)	1 (33.33)	11 (37.93)	6 (40.00)	15 (53.57)	0 (0)
Drop-in or unplanned support services (no appointment required)	88 (26.35)	2 (15.38)	14 (56.00)	1 (33.33)	16 (55.17)	3 (20.00)	17 (60.71)	3 (4.41)
Online support services	30 (8.98)	2 (15.38)	2 (8.00)	1 (33.33)	0 (0)	2 (13.33)	4 (14.29)	0 (0)
Onward referral to specialist bereavement support services	130 (38.92)	5 (38.46)	16 (64.00)	0 (0)	12 (41.38)	8 (53.33)	17 (60.71)	12 (17.65)
Supervision or support for staff	88 (26.35)	5 (38.46)	5 (20.00)	3 (100.00)	6 (20.69)	6 (40.00)	8 (28.57)	2 (2.94)
Staff training or education (internally or externally)	87 (26.05)	4 (30.77)	6 (24.00)	0 (0)	5 (17.24)	6 (40.00)	8 (28.57)	7 (10.29)
Other	29 (8.68)	4 (30.77)	1 (4.00)	0 (0)	0 (0)	0 (0)	3 (10.71)	10 (14.71)

	**Country**							
	**Netherlands (*n* = 5)**	**Northern Ireland (*n* = 13)**	**Republic of Ireland (*n* = 16)**	**Slovakia (*n* = 21)**	**Spain (*n* = 6)**	**Sweden (*n* = 17)**	**US (*n* = 66)**	**Other (*n* = 9)**
Immediate post-death support	2 (40.00)	6 (46.15)	13 (81.25)	17 (80.95)	5 (83.33)	15 (88.24)	43 (65.15)	7 (77.78)
Sending information to bereaved individuals / families	3 (60.00)	12 (92.31)	15 (93.75)	4 (19.05)	3 (50.00)	5 (29.41)	43 (65.15)	4 (44.44)
Group support for adults	1 (20.00)	4 (30.77)	6 (37.50)	1 (4.76)	0 (0)	2 (11.76)	19 (28.79)	5 (55.56)
Group support for children	0 (0)	1 (7.69)	5 (31.25)	0 (0)	1 (16.67)	1 (5.88)	5 (7.58)	4 (44.44)
Home visits	1 (20.00)	7 (53.85)	8 (50.00)	8 (38.10)	1 (16.67)	9 (52.94)	10 (15.15)	5 (55.56)
Remembrance services	2 (40.00)	2 (15.38)	11 (68.75)	9 (42.86)	2 (33.33)	1 (5.88)	19 (28.79)	4 (44.44)
Drop-in or unplanned support services (no appointment required)	2 (40.00)	3 (23.08)	2 (12.50)	5 (23.81)	1 (16.67)	3 (17.65)	13 (19.70)	3 (33.33)
Online support services	1 (20.00)	2 (15.38)	2 (12.50)	5 (23.81)	1 (16.67)	5 (29.41)	2 (3.03)	1 (11.11)
Onward referral to specialist bereavement support services	0 (0)	11 (84.62)	8 (50.00)	5 (23.81)	2 (33.33)	2 (11.76)	29 (43.94)	3 (33.33)
Supervision or support for staff	1 (20.00)	3 (23.08)	8 (50.00)	4 (19.05)	0 (0)	4 (23.53)	29 (43.94)	4 (44.44)
Staff training or education (internally or externally)	1 (20.00)	5 (38.46)	8 (50.00)	3 (14.29)	1 (16.67)	4 (23.53)	24 (36.36)	5 (55.56)
Other	0 (0)	0 (0)	2 (12.50)	1 (4.76)	1 (16.67)	0 (0)	7 (10.61)	0 (0)

Note. Reported are valid column percentages to account for missing data. Multiple response options were allowed.

Survey questionnaire (English language version)

What is your age? ____

- ○ Prefer not to say

What gender do you identify as?

- ○ Male
- ○ Female
- ○ Other
- ○ Prefer not to say

What is the highest degree or level of education you have completed?

- ○ Bachelor's Degree
- ○ Master's Degree
- ○ Ph.D.
- ○ Other
- ○ Prefer not to say

What country do you work in?

- ○ Australia
- ○ Austria
- ○ Czech Republic

- ○ England
- ○ Germany
- ○ Hong Kong
- ○ Hungary
- ○ Ireland (Northern)
- ○ Ireland (Republic)
- ○ Italy
- ○ Netherlands
- ○ New Zealand
- ○ Portugal
- ○ Scotland
- ○ Slovakia
- ○ Spain
- ○ Sweden
- ○ United States of America
- ○ Wales
- ○ Other ____

What is your current employment status?

- ◯ Employed Full-Time
- ◯ Employed Part-Time
- ◯ Self-employed
- ◯ Unemployed
- ◯ Retired
- ◯ Prefer not to say

Where did you work <u>during the COVID-19 pandemic</u>? ***Tick all that apply:***

	Adults	Children
Volunteer Hospice Service	☐	☐
Home Palliative Care Service	☐	☐
Inpatient Hospice	☐	☐
Hospital Palliative Care Support Team	☐	☐
Palliative Care Unit	☐	☐
Day Hospice, Day Care Centre	☐	☐
Other	☐	☐

How many years have you worked in palliative care social work?

____

Who did you provide palliative care social work services to <u>before the COVID-19 pandemic</u>? *Tick all that apply:*

- ☐ Children with a life-limiting illness
- ☐ Young people with a life-limiting illness
- ☐ Adults with a life-limiting illness
- ☐ Family carers of patients with a life-limiting illness
- ☐ Bereaved relatives or friends
- ☐ Staff support/supervision for social workers
- ☐ Staff support/supervision for other member of the MDT
- ☐ Staff training internally
- ☐ Education externally
- ☐ Fundraising
- ☐ Public relations
- ☐ Other - Please specify: ____

<u>Before COVID-19</u>, what support did you provide after the death of patients? *Tick all that apply:*

- ☐ Immediate post-death support
- ☐ Sending information to bereaved individuals/families
- ☐ Group support for adults
- ☐ Group support for children
- ☐ Home visits
- ☐ Remembrance services
- ☐ Drop-in or unplanned support services (no appointment required)
- ☐ Online support services
- ☐ Onward referral to specialist bereavement support services
- ☐ Supervision or support for staff
- ☐ Staff training or education (internally or externally)
- ☐ Other - Please specify: ____

Before COVID-19, approximately how many referrals would your social work service usually receive each month?

- ◯ 0 - 10
- ◯ 11 - 20
- ◯ 21 - 30
- ◯ 31 - 40
- ◯ 41 - 50
- ◯ 51 - 60
- ◯ 61 - 70
- ◯ 71 - 80
- ◯ 81 - 90
- ◯ 91 - 100
- ◯ 101+

During COVID-19, has the number of referrals for social work support changed?

- ◯ Lower than before COVID-19
- ◯ About the same
- ◯ A little higher than before COVID-19
- ◯ Much higher than before COVID-19

How did COVID-19 affect you or other staff involved in providing palliative care social work services or bereavement support in your organisation? *Tick all that apply:*

- ☐ Social work staff were furloughed
- ☐ Social work staff worked from home
- ☐ Social work staff were made redundant
- ☐ Social work staff redeployed
- ☐ Intensity of the work has changed
- ☐ Fundraising events were cancelled or postponed
- ☐ New fundraising events/appeals were introduced
- ☐ Higher levels of staff absence due to illness
- ☐ Higher levels of staff absence due to caring for dependents
- ☐ Increased pressure on staff due to increased number of clients
- ☐ Increased pressure on staff due to additional duties
- ☐ Increased pressure on staff due to nature of COVID-19 restrictions
- ☐ Purchasing new IT equipment to work remotely
- ☐ Using IT for online meetings/appointments
- ☐ Educating patients/carers for online meetings/appointments
- ☐ Facilitating communication online between patients and families/friends
- ☐ Increased need for supervision or staff support
- ☐ Positive team dynamics/cohesion
- ☐ Better work-life balance

- [ ] Increased understanding of the social work role
- [ ] Increased respect of the social work role
- [ ] Other - Please specify: ____

How has COVID-19 affected the social work support you provide? ***Tick all that apply:***

- [ ] We could no longer deliver the full range of social work services - Please describe here: ____
- [ ] We changed existing social work services - Please describe here: ____
- [ ] We introduced new social work services - Please describe here: ____
- [ ] None of the options
- [ ] Other changes were made that affected your services or support - Please specify: ____

What strategies did you develop as a team or within your organisation for self-care or mutual support?

____

____

____

____

____

Do you think COVID-19 has led to any positive changes or opportunities for social work services?

○ No

○ Yes - Please describe below: ____

Please tell us more about changes to your social work role due to the COVID-19 pandemic.

____

____

____

____

____

Do you think changes to your role or to the delivery of services were appropriate?

○ Yes - Comment: ____

○ No - Comment: ____

In hindsight, would you as a social worker have done anything different during the COVID-19 pandemic?

____

____

____

____

____
